# Primary hyperparathyroidism caused by enormous unilateral water-clear cell parathyroid hyperplasia

**DOI:** 10.1186/s12902-017-0207-1

**Published:** 2017-09-09

**Authors:** Georgios Boutzios, Helen Sarlanis, Anna Kolindou, Antigoni Velidaki, Theodore Karatzas

**Affiliations:** 1 Endocrine Unit, Department of Pathophysiology, National and Kapodistrian University of Athens, Medical School, Laiko General Hospital, Mikras Asias 75, 115 27 Athens, Greece; 2 Department of Nuclear Medicine, Laiko General Hospital, Athens, Greece; 3 Department of Pathology, National and Kapodistrian University of Athens, Medical School, Athens, Greece; 4 Second Department of Propedeutic Surgery, National and Kapodistrian University of Athens, Medical School, Laiko General Hospital, Athens, Greece

**Keywords:** Primary hyperparathyroidism, Water clear cell hyperplasia

## Abstract

**Background:**

Parathyroid water-clear cell hyperplasia (WCCH) and water-clear cell adenoma (WCCA) are rare causes of primary hyperparathyroidism. The frequency of WCCH seems to be less than 1% of all primary hyperplasia.

**Case presentation:**

We report a 53-year-old woman with a large unilateral water clear cell parathyroid hyperplasia associated with primary hyperparathyroidism and severe osteoporosis. Ultrasonography showed a 5.4 cm multilobulated hypoechoic well defined mass localized in the lower half of the left thyroid lobe. Technetium sestamibi scanning showed a persistent very large area of increased activity possibly corresponding to a left inferior double parathyroid adenoma. At surgery, two large merged lobulated parathyroid glands were removed from the left superior and inferior aspects of the adjacent thyroid extending to the sub-clavicular area. Histopathology showed polygonal hyperplastic vacuolated cells with abundant water clear cytoplasm. The lesion had lack of capsule or rim of parathyroid tissue and immunohistochemistry was positive for PTH staining. These findings were consistent with diffused water clear cell hyperplasia. After parathyroidectomy, iPTH and calcium levels dropped immediately.

**Conclusion:**

The clinical presentation of the patients with water clear cells parathyroid content and hyperparathyroidism is indistinguishable from that of the more common causes of primary hyperparathyroidism of adenoma or hyperplasia and the diagnosis is made only on pathological examination. In conclusion**,** the distinction of water clear cell hyperplasia from water clear cell adenoma can be challenging in many cases, although clinically significant as far as treatment and follow-up.

## Background

Primary hyperparathyroidism is a common endocrine disorder with an incidence of 21.6 per 100,000 person/year. Parathyroid water-clear cell hyperplasia (WCCH) and water-clear cell adenoma (WCCA) are rare causes of primary hyperparathyroidism, with hyperplasia to be more common than adenoma [1, 2]. The frequency of WCCH seems to be less than 1% of all primary hyperplasia [3]. It is characterized by an increase of the upper glands and may reach large sizes before involvement of the inferior ones [2, 3]. The classic form of WCCH involves all four glands, with finely vacuolated chief cells, thin or lack of capsule, and normal parathyroid tissue within or outside it. Water-clear cells (WCCs) are not part of the composition of normal human parathyroid glands and their presence has been associated with parathyroid hyperfunction [2, 3]. Also, WCCs represent transformed chief cells that frequently appear with advancing age [3, 4].

## Case presentation

**Fig. 1:**
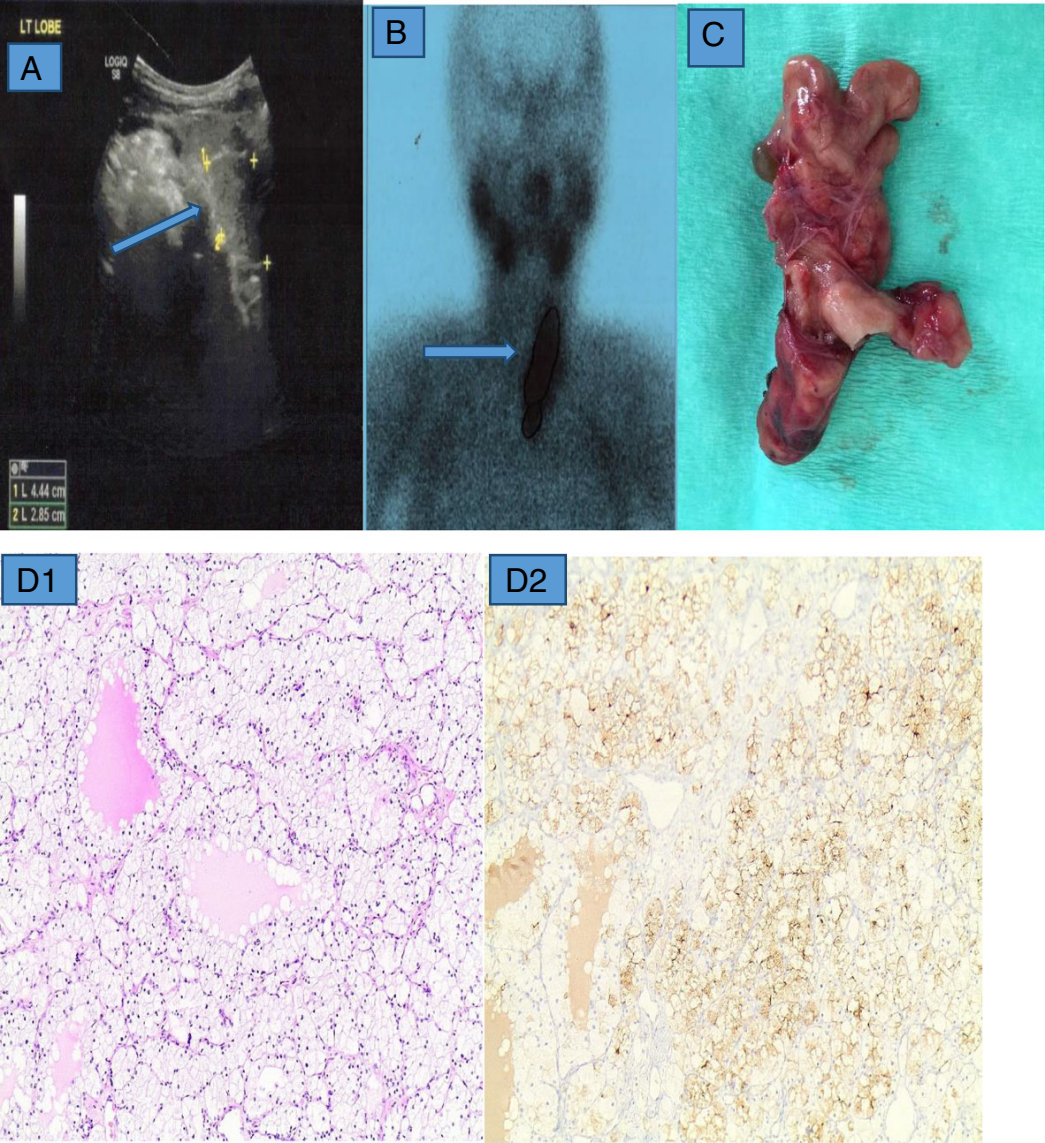
**a** Ultrasonography showed a 5.4 cm multilobulated hypoechoic well defined mass, localized in the lower half of the left thyroid lobe and submersible into the sub-clavicular area. The right parathyroid glands were within normal size. **b** Technetium (^99m^Tc) sestamibi scanning revealed a persistent large area of increased activity corresponding to a left inferior double parathyroid adenoma. **c** Surgical specimen: A multilobulated parathyroid mass. **d** Histopathology examination: Water clear-cell parathyroid hyperplasia: Distinct cell membranes demarcating abundant water-clear cytoplasm, ranging from granular to finely vacuolated. Small and normal nuclei, sometimes polar and oriented towards adjacent stroma and blood vessels. **d1** Hematoxylin and eosin, magnification 100X. **d2** PTH staining, magnification 100X

We herein report a case of primary hyperparathyroidism in a 53-year-old woman with multinodular goiter. The patient was referred to our endocrine outpatient clinic for assessment of persistently elevated serum calcium and severe osteoporosis. She was asymptomatic and she had no previous history of nephrolithiasis. She had a non-contributory past medical history and reported no family history of multiple endocrine neoplasia type 1. Biochemical analysis revealed corrected serum calcium of 11.1 mg/dl (reference range 8.4–10.2), serum phosphorus of 2.6 mg/dl (reference range 2.3–4.7), 25-OH vitamin D3 of 9.1 ng/ml (reference range 28–47), serum creatinine of 0.7 mg/dl (reference range 0.6–1.2) and an intact parathyroid hormone (iPTH) level of 398 pg/ml (reference range 10–65). The 24-h urine collection for calcium was 246 mg/24 h (reference range 100–300). Ultrasonography showed a 5.4 cm multilobulated hypoechoic well defined mass localized in the lower half of the left thyroid lobe and submersible into the sub-clavicular area. The right parathyroid glands were within normal size (Fig. 1a). Technetium (^99m^Tc) sestamibi scanning was consistent with a persistent very large area of increased activity possibly corresponding to a left inferior double parathyroid adenoma (Fig. 1b). The elevated preoperative levels of serum calcium and iPTH were not proportionate to the huge parathyroid mass. The patient underwent a minimally invasive parathyroidectomy. At surgery, two large merged lobulated, tan-colored parathyroid glands measuring 5.4 cm were removed from the left superior and inferior aspects of the adjacent thyroid extending inferiorly to the sub-clavicular area. The recurrent laryngeal nerve was coursing behind the mass and was carefully preserved. The right side was not explored. At surgery was not possible to distinguish whether the parathyroids represented a single hyperplastic gland or two adjacent adenomas that had coalesced (Fig. 1c). Histopathology revealed polygonal hyperplastic and/or hypertrophic cells, with a diffuse (solid sheet-like) pattern or arranged in acini, follicles, papillae or trabeculae, on occasion lining admixed microcystic degenerate formations containing proteinaceous material. Moreover, revealed distinct cell membranes demarcating abundant water clear cytoplasm, ranging from granular to finely vacuolated and also small and normal nuclei, sometimes polar and oriented towards adjacent stroma and blood vessels. Also, the lesion exhibited lobulated periphery with a lack of capsule or rim of parathyroid tissue (Fig. 1d1). Immunohistochemistry showed that the cells were positive for PTH staining (Fig. 1d2). These findings were consistent with diffused water clear cell hyperplasia. After parathyroidectomy iPTH dropped immediately to 54 pg/ml. The patient made an uneventful recovery, and 1 week postoperatively serum iPTH and calcium levels were 60 pg/ml, and 8.8 mg/dl respectively. Calcium carbonate plus 1a–vitamin D (alfacalcidol) supplementations were administered. The CARE guidelines were followed in this case.

## Discussion and conclusions

A rare cause of primary hyperparathyroidism is associated with the presence of water clear cells in the human parathyroid glands. The parathyroid glands have water clear cells content as their dominant histology. On microscopic examination, normal parathyroid tissue is replaced with growth of WCCs which have foamy cytoplasm containing vacuoles, instead of the chief cells of parathyroid hyperplasia or adenomas [5]. The clinical presentation of the patients with WCCs parathyroid hyperfunction is indistinguishable from that of the more common causes of primary hyperparathyroidism of adenoma or hyperplasia and the diagnosis is made only on pathological examination.

Water clear cell hyperplasia in it’s classic form involves all four glands by distinctive, finely vacuolated chief cells, thin capsule and normal parathyroid tissue neither within nor outside the gland. Distinction between water clear cell hyperplasia and adenoma may not always be possible owing to incomplete gland replacement by clear cells in hyperplasia or the presence of asymmetrical hyperplasia [6]. In WCCH the individual parathyroids may vary considerably in size and may not all be completely replaced by water clear cells. Most WCCAs are solitary, although rarely they may be multiple. The presence of an intervening fibrous capsule suggests an adenoma [2–4].

Typically, WCCH starts in the superior parathyroid glands, which may reach large sizes before involvement of the inferior glands [3, 4, 7]. This may occurred in our patient, although coalescence between both superior and inferior glands cannot be excluded. In our case, intra-operatively the contralateral side had not been explored as the pre-operative diagnosis was that of an adenoma. On the other hand, it was extremely difficult to establish the diagnosis during surgery of the large parathyroid tumors, based on the frozen section specimens, even though it was very important to define the histological type as adenoma or hyperplasia to consider the appropriate surgical excision.

In the present case, iPTH level was preoperatively highly elevated whereas serum calcium was not particularly high. Post-operatively, an immediate drop of iPTH and serum calcium levels was observed and remained within normal range until the last follow-up appointment 6 months later. A longer follow-up period (on a 6-month basis for at least 5 years) it is advised to our patient since the contralateral side had not been explored. A decision to monitor the patient rather than re-operate was taken based on clinical and biochemical cure.

In conclusion, WCCH is a very rare cause of primary hyperparathyroidism. Its distinction from WCCA can be challenging in many cases, although clinically significant concerning treatment and follow-up. However, the histological appearance of both entities is markedly evident. In our case, preoperative imaging showed an asymmetrical enlargement of bilobular mass of the left inferior parathyroid gland and normal glands in the contralateral side, leading to a unilateral approach. A strict follow-up period will be continued since the possibility of contralateral recurrence cannot be excluded.

## Abbreviations

iPTH: Intact parathyroid hormone
WCC: Water-clear cells
WCCA: Water-clear cell adenoma
WCCH: Water-clear cell hyperplasia
